# Discovery of urinary biosignatures for tuberculosis and nontuberculous mycobacteria classification using metabolomics and machine learning

**DOI:** 10.1038/s41598-024-66113-x

**Published:** 2024-07-03

**Authors:** Nguyen Ky Anh, Nguyen Ky Phat, Nguyen Quang Thu, Nguyen Tran Nam Tien, Cho Eunsu, Ho-Sook Kim, Duc Ninh Nguyen, Dong Hyun Kim, Nguyen Phuoc Long, Jee Youn Oh

**Affiliations:** 1 Department of Pharmacology and PharmacoGenomics Research Center, Inje University College of Medicine, Busan, 47392 Republic of Korea; 2 Faculty of Pharmacy, Ton Duc Thang University, Ho Chi Minh City, Vietnam; 3 Section for Comparative Pediatrics and Nutrition, Department of Veterinary and Animal Sciences, University of Copenhagen, 1870 Frederiksberg, Denmark; 4 Division of Pulmonary, Allergy and Critical Care Medicine, Department of Internal Medicine, Korea University Guro Hospital, Seoul, 08308 Republic of Korea

**Keywords:** Nontuberculous mycobacteria, Tuberculosis, Differential diagnosis, Diagnostic biomarkers, Metabolomics, Machine learning, Diagnostic markers, Molecular medicine, Tuberculosis

## Abstract

Nontuberculous mycobacteria (NTM) infection diagnosis remains a challenge due to its overlapping clinical symptoms with tuberculosis (TB), leading to inappropriate treatment. Herein, we employed noninvasive metabolic phenotyping coupled with comprehensive statistical modeling to discover potential biomarkers for the differential diagnosis of NTM infection versus TB. Urine samples from 19 NTM and 35 TB patients were collected, and untargeted metabolomics was performed using rapid liquid chromatography-mass spectrometry. The urine metabolome was analyzed using a combination of univariate and multivariate statistical approaches, incorporating machine learning. Univariate analysis revealed significant alterations in amino acids, especially tryptophan metabolism, in NTM infection compared to TB. Specifically, NTM infection was associated with upregulated levels of methionine but downregulated levels of glutarate, valine, 3-hydroxyanthranilate, and tryptophan. Five machine learning models were used to classify NTM and TB. Notably, the random forest model demonstrated excellent performance [area under the receiver operating characteristic (ROC) curve greater than 0.8] in distinguishing NTM from TB. Six potential biomarkers for NTM infection diagnosis, including methionine, valine, glutarate, 3-hydroxyanthranilate, corticosterone, and indole-3-carboxyaldehyde, were revealed from univariate ROC analysis and machine learning models. Altogether, our study suggested new noninvasive biomarkers and laid a foundation for applying machine learning to NTM differential diagnosis.

## Introduction

Nontuberculous mycobacteria (NTM) is a group of environmental bacteria consisting of more than 190 species and subspecies of *Mycobacterium*, except for *Mycobacterium tuberculosis (Mtb)* and *Mycobacterium leprae*^1^. NTM infection is an opportunistic disease with a wide range of virulence due to its species diversity^2^. The incidence of NTM pulmonary disease has increased in the last decade and is becoming a global health crisis^3,4^. In a systematic review and meta-analysis study conducted in 2022 using global, culture-based, microbiologic data from 2000, the worldwide overall increase rate of NTM infection was found to be 4.1% per year^5^. While curable, treating NTM infection requires a prolonged multidrug regimen with restricted options and significant risks of antibiotic resistance, reinfection, or relapse^1,2,6^. Therefore, the initiation of NTM treatment should be carefully considered and guided by an accurate diagnosis. However, clinical manifestations, smear microscopy, and medical imaging of NTM pulmonary disease and pulmonary tuberculosis (TB) largely overlap^7–9^. This may lead to misdiagnosis of NTM infection as TB. The current gold standard for confirming NTM infection is mycobacterial culture, a time-consuming process^2,9^. Except for fast growers, a majority of NTM species require 2–3 weeks to grow in subculture^9,10^. These challenges prevent the timely diagnosis of NTM infection. Taken together, a quick and accurate diagnostic method is essential to overcome these difficulties and support the decision to initiate NTM treatment.

Advances in molecular diagnosis have allowed faster detection of NTM infection. Polymerase chain reaction (PCR)-based assays are the most widely used methods^11–13^. For example, the Cepheid Xpert MTB/RIF assay can differentiate *Mtb* from NTM^14,15^. Its negative result combined with a positive result on acid-fast bacilli smear microscopy can indicate the presence of NTM^14,16^. However, Xpert MTB/RIF has a low sensitivity in paucibacillary specimens and the risk of misdiagnosing NTM as *Mtb* in a high bacterial load setting^16–18^. Compared to Xpert MTB/RIF, multiplex PCR sequencing of *hsp65*, *rpoB*, as well as 16S rRNA allows the fast detection of NTM with better specificity and identification of the NTM species in the sample^2,16,19^. PCR-based tests and mycobacterial culture often utilize sample types such as sputum, bronchial wash, or bronchial lavage. The collection of these samples can be invasive or complicated, particularly for patients in the early stages of infection^20^. Since NTM is naturally present in the environment, it can be challenging to distinguish an NTM-contaminated sample from a sample with an actual NTM infection using PCR-based tests^2,16^. Overall, there is still room for developing new diagnostic tests using easily accessible samples to improve healthcare quality for NTM patients.

The emergence of host-based biomarker research may introduce a novel approach to NTM diagnosis^21–25^. Metabolomics is one of the most commonly employed methods for identifying new metabolic biomarkers of infectious diseases^23,26^. Furthermore, various biological fluids, including blood and urine, can be employed for metabolomic analysis^27^. This versatility warrants the development of diagnostic tests based on readily accessible samples. Previous studies have demonstrated the potential of using metabolomics approach to identify biomarkers for TB diagnosis^18,23,28^. Investigating the metabolic profile of NTM and TB may shed light on the metabolic features that could differentiate NTM pulmonary disease from TB. To the best of our knowledge, there have been no documented reports on metabolic biomarkers that distinguish between TB and NTM infection. Addressing this research gap may open the opportunity to better understand the metabolic alteration underlying these two infections and develop novel diagnostic tools.

A urine-based assay has several advantages in clinical settings, especially as a triage test. Firstly, urine sampling is non-invasive, and patients can conveniently collect it. Secondly, urine is easy to store. Thirdly, urine is less hazardous than sputum, thus minimizing the need for biosafety measures^18,29^. Moreover, urine is a rich source of metabolites, making it an appealing sample type for metabolomics analysis^30^. Nevertheless, the concentration and composition of metabolites may vary significantly between urinary samples due to hydration levels or other factors such as diet, kidney diseases, and medications. This challenges metabolomics data interpretation and requires a standardized approach with careful technical considerations that can handle the variability in urinary samples^31^. An automated platform could control laboratory variability, reduce delays, and lower the cost of analyzing results^16,18,32^. The application of machine learning (ML), which is easy to automate, scale up, and update, for data analysis, can aid the establishment of an automated diagnostic system^33^. In addition, ML is excellent in data mining, especially for handling complex relationships towards prediction. This characteristic makes ML suitable for omics-based large-scale, high-dimensional datasets^34,35^. ML has also gained attention for improving the accuracy of clinical diagnosis^35,36^. Indeed, ML has been used to accurately distinguish between NTM pulmonary disease and pulmonary TB (using CT image data)^37^. Finally, ML can prioritize important biomarker candidates by calculating the feature importance score^38^.

This study aims to employ an ML-assisted metabolomics approach to investigate the differences in urinary metabolic profiles between patients with NTM pulmonary disease and those with TB. By investigating these differences, we seek to identify promising biomarker candidates that can classify NTM and TB patients. The findings from this work may establish a foundation for the development of non-invasive, urine-based diagnostic tests for NTM pulmonary disease to facilitate timely and appropriate treatment interventions.

## Materials and methods

### Study design and sample characteristics

**Figure 1:**
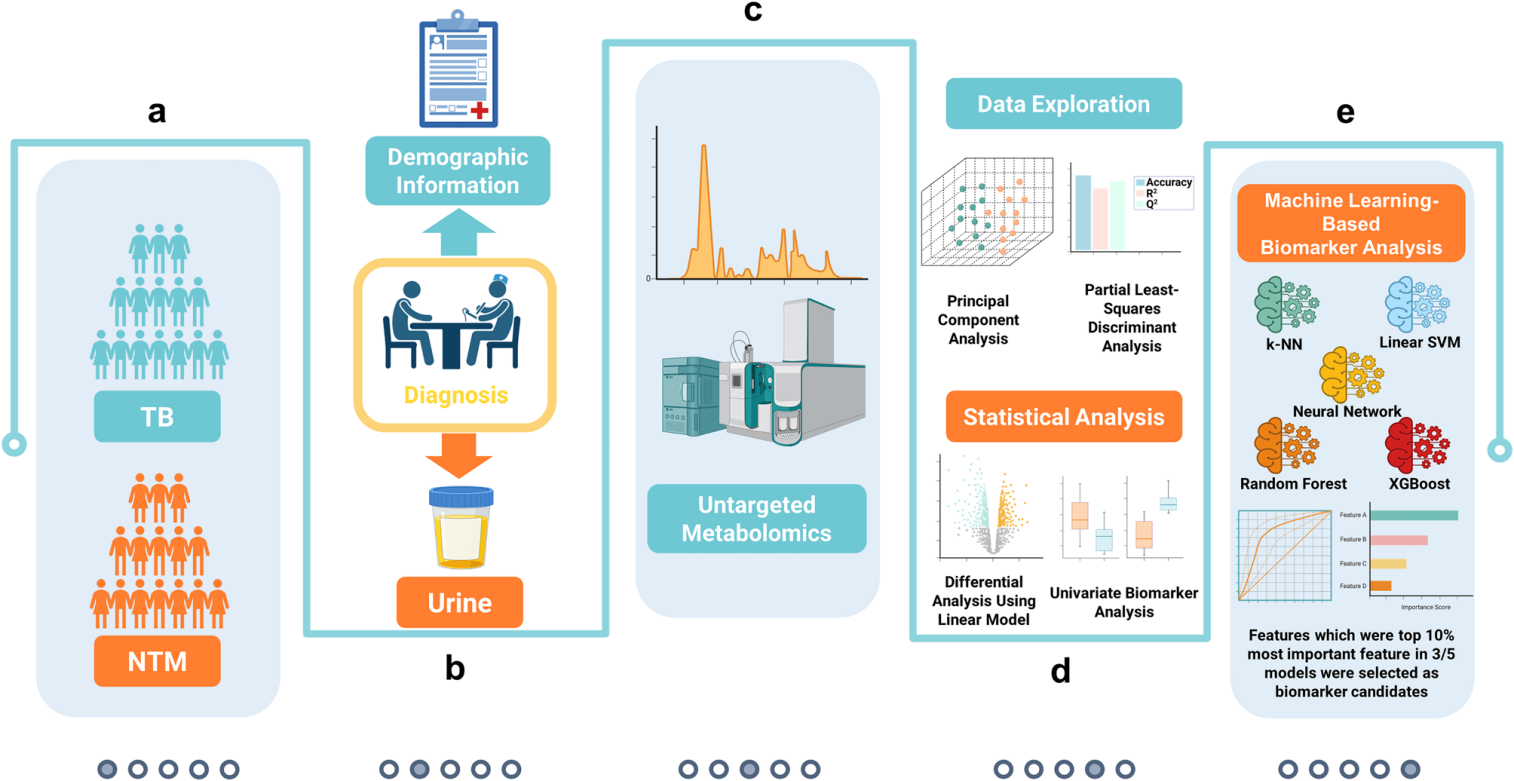
Workflow of the study. (**a**) Subject enrollment. (**b**) Sample collection. (**c**) Untargeted metabolic profiling. (**d**) Conventional statistical analysis-based biomarker identification. (**e**) Machine learning-assisted biomarker discovery. *NTM* nontuberculous mycobacteria, *TB* tuberculosis, *k-NN* k-nearest neighbors, *SVM* support vector machine.

The clinical study, which included urine samples and information of participants, was reviewed and approved by the Institutional Review Board of Guro Hospital, Korea University (No. 2017GR0012). All procedures were carried out in accordance with the Declaration of Helsinki. Written informed consent was obtained from all participants prior to study procedures. The diagnosis of TB followed the World Health Organization guideline for drug-susceptible TB, which considered clinical symptoms, sputum smear microscopy and culture tests, radiological examination, and GeneXpert MTB/RIF assay. The diagnosis of NTM infection was based on the clinical practice guideline of the American Thoracic Society, European Respiratory Society, European Society of Clinical Microbiology and Infectious Diseases, and Infectious Diseases Society of America^1^. Briefly, patients were classified as NTM infection according to clinical pulmonary and systemic symptoms, radiographic information, and microbiological criteria. For radiographic information, patients were considered NTM infection if they had nodular or cavitary opacities on chest radiograph or multifocal bronchiectasis with multiple small nodules on high-resolution computed tomography scan. For microbiological criteria, sputum culture result and mycobacterial histologic features were considered. Patients with NTM should meet at least one of the three criteria as described by the guideline. Patients with latent TB infection, malignant conditions, or who were receiving immunosuppressive therapy were excluded. The current study included 54 patients over 18 years of age, with 19 patients in the NTM infection and 35 patients in the control group diagnosed with pulmonary TB (Table 1). Urine samples were collected before patients received antimicrobial treatment. The workflow of this study is depicted in Fig. 1.

**Table 1:** Clinical characteristics of the subjects.

	TB group (N = 35)	NTM group (N = 19)	P-value
Age, years	57 (45–65)	67 (59–75)	0.0024
BMI, kg.m^-2^	21.60 (19.20–23.35)	20.60 (19.45–22.65)	0.7788
Sex			
Male	25 (71)	6 (32)	0.0087
Female	10 (29)	13 (68)	
Comorbidity			
Diabetes	6 (17)	0 (0)	0.0797
Hypertension	7 (20)	6 (32)	0.5061
NTM classification			
*M. avium*	–	6 (32)	
*M. intracellulare*	–	13 (68)	
Radiographic findings			
Cavities	3 (9)	4 (21)	
No cavity	32 (91)	15 (79)	
Smoking status			
Never	13 (37)	15 (79)	0.0045
Former	14 (40)	-	
Current	8 (23)	4 (21)	
Alcohol consumption			
Yes	20 (57)	3 (16)	
No	15 (43)	14 (74)	
Unknown	–	2 (10)	

Data are presented as N (%) or median (interquartile range). For P-value calculation, Wilcoxon rank-sum test was used for continuous variables while Fisher’s exact test was used for categorical variables. A P-value less than 0.05 was considered statistically significant.

### Chemicals, reagents, and consumables

Liquid chromatography﻿–mass spectrometry (LC–MS) grade acetonitrile (ACN), formic acid, methanol (MeOH), and water were obtained from Sigma-Aldrich (St. Louis, Missouri, USA). Six internal standards for metabolomics, namely, ﻿L-tryptophan-(indole-d5), acetyl-L-carnitine-(N-methyl-d3), cholic acid-2,2,3,4,4-d5, L-phenyl-d5-alanine, leucine enkephalin, and SM(d18:1/15:0)-d9 were provided by Sigma–Aldrich (St. Louis, Missouri, USA). ACQUITY UPLC HSS T3 pre-column (5 × 2.1 mm; 1.8 µm particle size) and ACQUITY UPLC HSS T3 column (50 × 2.1 mm; 1.8 µm particle size) were purchased from Waters (Milford, MA, USA).

### Sample preparation

The first urine in the morning of each patient was collected for the study. Then, the samples were stored at − 80 °C prior to the metabolite extraction. Urease pre-treatment was not performed as it might introduce artifacts into the metabolites^39^. Metabolites from urine samples were extracted as follows. First, 50 µL of each urine sample was thawed on ice for approximately 30 min. The samples were vortexed briefly for 10 s. For protein precipitation, 150 µL of MeOH (kept at − 80 °C) containing six internal standards, namely, L-tryptophan-(indole-d5), acetyl-L-carnitine-(N-methyl-d3), cholic acid-2,2,3,4,4-d5, L-phenyl-d5-alanine, leucine enkephalin, and SM(d18:1/15:0)-d9, was added to the samples. Then, the mixtures were vortexed vigorously for 30 s and centrifuged for 2 min at 14,000 *rcf* and 4 °C. Next, 150 µL of the supernatant from each mixture was evaporated under a flow of nitrogen at room temperature. The dried residues were kept briefly at − 20 °C and used for subsequent analysis.

### Untargeted metabolomics

The dried extracts were redissolved in 200 µL of 50% MeOH for untargeted metabolomics. The mixtures were then vortexed and centrifuged at 14,000 *rcf* and 4 °C for 2 min. One hundred and fifty microliters of supernatant from each sample were used for subsequent analysis while 20 µL was taken for pooled quality control (QC) sample. The samples were kept at 4 °C in an autosampler prior to LC–MS analysis. A Shimadzu Nexera UPLC system (Kyoto, Japan) coupled with an X500R Quadrupole time-of-flight mass spectrometer (SCIEX, MA, USA) was utilized to separate and analyze the urine metabolome. Separation was achieved by an ACQUITY UPLC HSS T3 column (50 × 2.1 mm; 1.8 µm particle size) coupled to an ACQUITY UPLC HSS T3 pre-column (5 × 2.1 mm; 1.8 µm particle size), with a gradient elution following established method^40^. The binary mobile phases consisted of (A) water with 0.2% formic acid and (B) ACN with 0.1% formic acid. The total run time of each sample was 5.5 min including the pre-injection step. For compound ionization, electrospray ionization (ESI) was employed. Information-dependent Acquisition mode was used to acquire the data. Injection volumes for positive ion (ESI+) mode and negative ion (ESI−) mode were set at 1.5 µL and 3 µL, respectively. To maintain the quality and consistency of the analysis, mass calibration was carried out after every eight injections using the X500R calibration solution by a calibrant delivery system.

### Data processing and alignment

MS-DIAL version 4.9.0 was utilized to preprocess the raw data (.wiff files)^41^. The parameters used for data processing followed our previous report^42^. An in-house library of more than 600 endogenous metabolites developed under the same analytical condition, taken from the mass spectrometry metabolite library of standards, was employed for metabolite annotation. Metabolites that matched the mass-to-charge ratio and retention time with the standards were annotated. Furthermore, the public libraries of MS-DIAL were used for MS/MS spectra inspection. Next, the aligned data were exported from MS-DIAL and were further processed using MetaboAnalystR version 4.0.0. First, features with a missing data rate of over 50% were removed. Then, the feature-wise k-nearest neighbors (k-NN) algorithm was used for missing value imputation^43^. Next, features with a relative standard deviation greater than 25% in the QC group and near-constant features were excluded. The filtered data were subjected to normalization using creatinine and quantile normalization.

### Data exploration and statistical analysis

Urinary metabolome data were explored using principal component analysis (PCA). For classification between the TB and NTM metabolomes, partial least squares–discriminant analysis (PLS-DA) was employed. The performance of the PLS–DA models was assessed using five-fold cross-validation, and the optimal model was chosen using Q^2^ value. PCA and PLS-DA analyses were conducted using MetaboAnalystR 4.0.0^44^. Normalized data were log-transformed and Pareto scaled prior to these analyses. The PCA and PLS-DA results were visualized using plotly (version 4.10.3).

Univariate analysis using linear models with covariate adjustment was conducted to discover differential metabolites (DMs) between NTM and TB. First, normalized data were log-transformed. Then, the age-, BMI- and sex-adjusted linear model was then used to identify DMs between the NTM and TB groups. A |fold change (FC)| of 1.5 and a false discovery rate (FDR) of 0.05 were applied as the thresholds for significantly altered metabolites. FDR was calculated using Benjamini–Hochberg procedure. Univariate receiver operating characteristic (ROC) analysis was applied to examine the ability of metabolites to differentiate between NTM infection and TB. Metabolites with an area under the curve (AUC) ≥ 0.7 and P-value < 0.05 were considered to have good performance in classifying the two groups. The univariate analyses were conducted using MetaboAnalyst 5.0^45^.

### ML models for biomarker identification

ML was used to classify NTM and TB and identify biomarkers that significantly contribute to the classification performance. The dataset used for ML-assisted biomarker discovery comprised non-overlapping (between two ion modes) normalized annotated metabolic features and demographic information (age, sex, BMI). Continuous variables were scaled using the standard scaler method (the training and test sets were scaled separately). In addition, categorical variables were one-hot encoded^46^. We considered five commonly used classification methods, including k-NN, linear support vector machine (SVM), random forest (RF), extreme gradient boosting (XGB), and neural network (NN). The five-fold nested cross-validation procedure was conducted for model validation, wherein the outer loop involved splitting the data into training and testing sets, while the inner loop focused on finding the optimal hyperparameters. The AUC value of the ROC curve was used to evaluate model performance. The caret package (version 6.0-94) was used in R version 4.3.2 for model building and validation^47^. To determine which variables are potential biomarker candidates, a voting strategy was applied. In detail, importance scores of variables were computed for each model. Then, variables were ranked in descending order based on the importance score. The top 10% of the most important variables for each model were selected and subjected to a Venn analysis. A variable was considered significant for distinguishing NTM from TB if the Venn analysis results showed that it ranked within the top 10% of variables based on importance scores for at least three models.

## Results

### Clinical characteristics of the study population

The study population (N = 54) consisted of 19 NTM patients infected with *Mycobacterium avium* complex and 35 TB patients. Regarding the NTM group, the median age was 67 [interquartile range (IQR) = 59–75] and median BMI was 20.60 (IQR = 19.45–22.65) kg.m^−2^. In the TB group, the median age was 57 (IQR = 45–65) and median BMI was 21.60 (IQR = 19.20–23.35) kg.m^−2^. In the NTM group, the percentage of females was 68%, while it was 29% in the TB group. There were significant differences in age (P-value = 0.0024) and sex ratio (P-value = 0.0087) between the two groups. Of note, 32% of the patients in the NTM group had hypertension, no patients had diabetes, and 21% were smokers at the time of diagnosis and sample collection. The percentage of patients with diabetes in the TB group was 17%, 20% had hypertension and 23% were current smokers (Table 1). Notably, there was a significant difference in the percentage of smoking status between the two groups.

### Multivariate models of urinary metabolomics data

**Figure 2:**
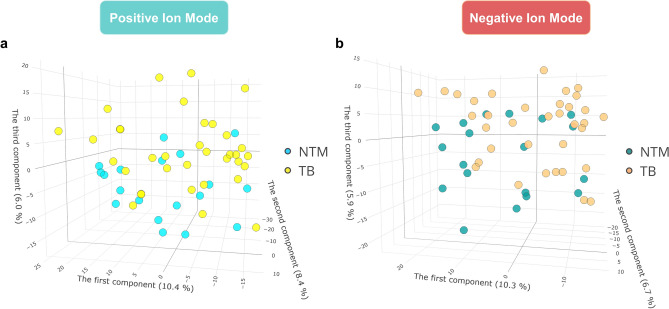
Principal components analysis scores plots of metabolome of NTM and TB patients. (**a**) Positive ion mode. (**b**) Negative ion mode. *NTM* nontuberculous mycobacteria, *TB* tuberculosis.

PCA was used to explore sample variance regardless of sample origin. In the PCA of all samples (including QC samples), the QC samples clustered tightly, as shown in the scores plots of both ESI+ mode and ESI− mode (Supplementary Fig. S1). The results indicated a consistent data acquisition process, which enabled subsequent analysis. In the PCA of NTM and TB samples, the scores plot of the ESI+ mode showed no apparent separation between the two groups (Fig. 2a). Consistent with the ESI+ mode, no clear separation between NTM and TB groups was observed in the ESI− mode scores plot (Fig. 2b). It is worth noting that in the analysis of both ion modes, the first two principal components only explained less than 20% of the variance in the data, implicating that the relationships between features were complex.

**Figure 3:**
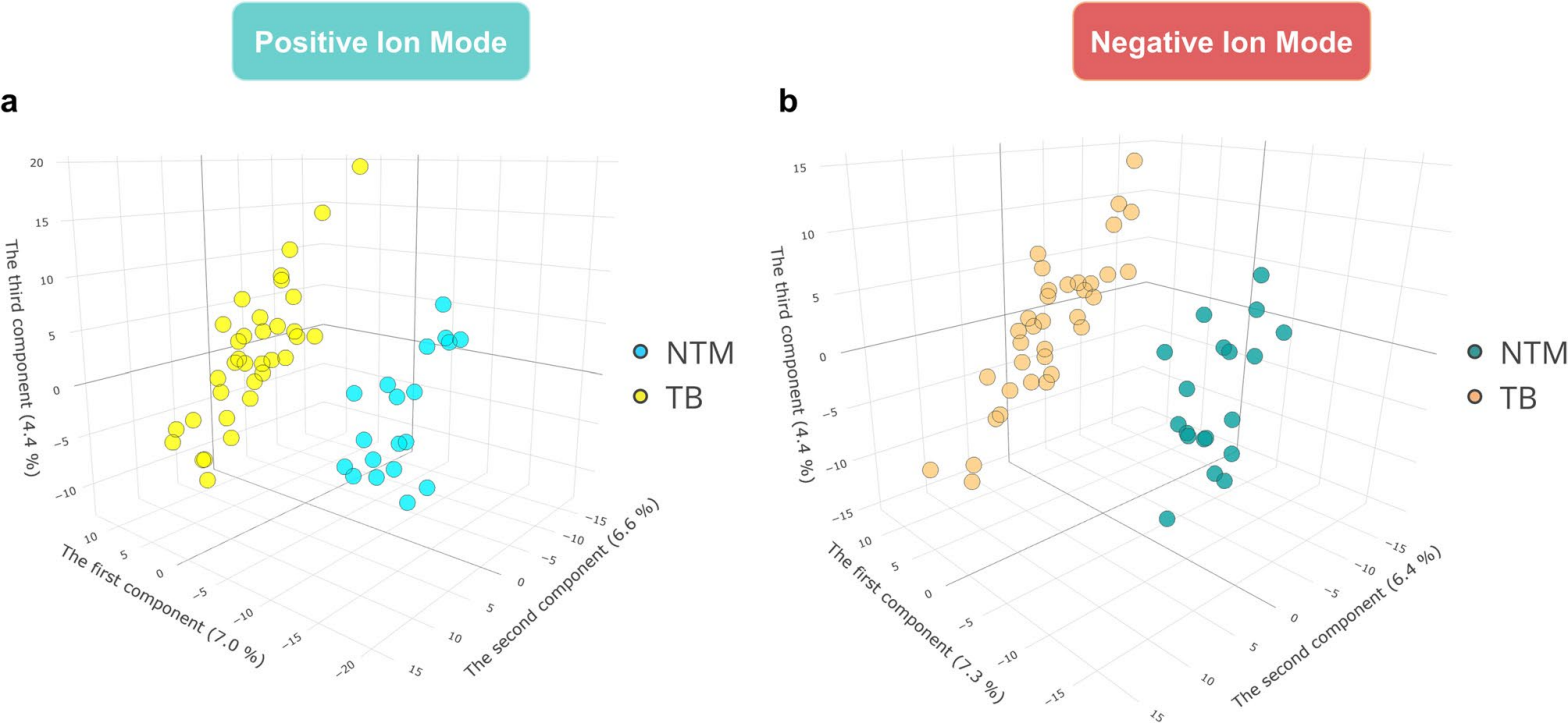
Partial least squares discriminant analysis scores plots of metabolome of NTM and TB patients. (**a**) Positive ion mode. (**b**) Negative ion mode. *NTM* nontuberculous mycobacteria, *TB* tuberculosis.

PLS-DA was conducted to differentiate the NTM and TB groups. In the ESI+ mode, the PLS-DA scores plot showed a separation between the two groups (Fig. 3a). However, the predictive performance of the model was limited, as indicated by the cross-validation process (accuracy = 0.72, R^2^ = 0.56, Q^2^ = 0.17) (Supplementary Fig. S2). The performance of the ESI − mode model was consistent with the ESI+ mode model (accuracy = 0.8, R^2^ = 0.81, Q^2^ = 0.18) as shown in Fig. 3b and Supplementary Fig. S2. Similar to PCA, the first and second principal components of the PLS-DA models explained less than 15% of the data variance.

To identify the DMs between NTM and TB groups, a linear model with clinical covariates adjustment was employed (Supplementary Fig. S3). In the analysis of the ESI+ mode, a total of 302 features had a P-value less than 0.05. Notably, the level of tetradecenoylcarnitine had a 2.7-fold increase in the NTM group compared to TB. Methionine, hypoxanthine, and acetylserotonin were also upregulated by 1.8-fold. In contrast, the level of 4-guanidinobutanoate had a nearly three-fold decrease while adenosine, cystine, and acetylcholine reduced by approximately 1.7-fold. However, no feature remained significant when considering the FDR criteria of less than 0.05. In the analysis of the ESI- mode, 287 features had a P-value < 0.05. Among them, levels of glutarate and tartrate increased 1.8- and 2.7-fold in the NTM group, respectively, while the level of 4-imidazoleacetate decreased 2.3-fold. Similar to the ESI+ mode, no feature qualified the FDR < 0.05 criteria in the ESI- mode analysis. The P-value and FDR of pre- and post-covariates-adjustment were depicted in Supplementary Fig. S4. Together, the univariate and multivariate analyses implicated the complexity of the data. Linear models might not be the optimal solution for identifying the differences between the NTM and TB groups.

### Univariate receiver operating characteristic curve analysis for biomarker discovery

Univariate ROC analysis was performed to identify potential biomarkers for the classification of NTM infection and TB (Table 2). In the ESI+ analysis, 9 annotated features had an AUC ≥ 0.7 and P-value < 0.05. Among them, valine, indole-3-carboxyaldehyde, and corticosterone demonstrated the best performance with an AUC of approximately 0.8. In the ESI− analysis, eight metabolites had an AUC greater than 0.7. Particularly, tryptophan and glutarate demonstrated a good performance with an AUC of approximately 0.8. Interestingly, the metabolites that showed good classification performance were amino acids such as valine, histidine, tyrosine, and tryptophan.

**Table 2:** Univariate receiver operating characteristic analysis of the biomarker candidates for nontuberculous mycobacteria.

Analyte	Mode	AUC	Fold change	P-value
Valine	POS	0.788	0.59	0.0009
Indole-3-carboxyaldehyde	POS	0.772	0.63	0.0015
Corticosterone	POS	0.762	0.66	0.0018
Methionine	POS	0.747	2.63	0.0020
Histidine	POS	0.739	0.54	0.0032
Acetylcholine	POS	0.726	0.57	0.0460
N-Acetylserotonin	POS	0.725	1.78	0.0074
Adenosine	POS	0.710	0.19	0.0250
Tyrosine^1^	POS	0.702	0.62	0.0189
Tryptophan	NEG	0.771	0.76	0.0049
Glutarate	NEG	0.765	1.22	0.0003
Oxoglutarate	NEG	0.725	1.35	0.0059
Caffeate	NEG	0.716	1.39	0.0071
Tyrosine^1^	NEG	0.710	0.85	0.0105
3-Hydroxyanthranilate	NEG	0.708	0.78	0.0133
Succinate	NEG	0.707	1.33	0.0365
3-Methoxytyrosine	NEG	0.706	0.66	0.0059

*POS* Positive ion mode, *NEG* Negative ion mode. Fold change is presented as the ratio of analyte abundance in the NTM group compared to the TB group.

^1^Metabolite detected in both ion mode.

### ML models for biomarker discovery

**Figure 4:**
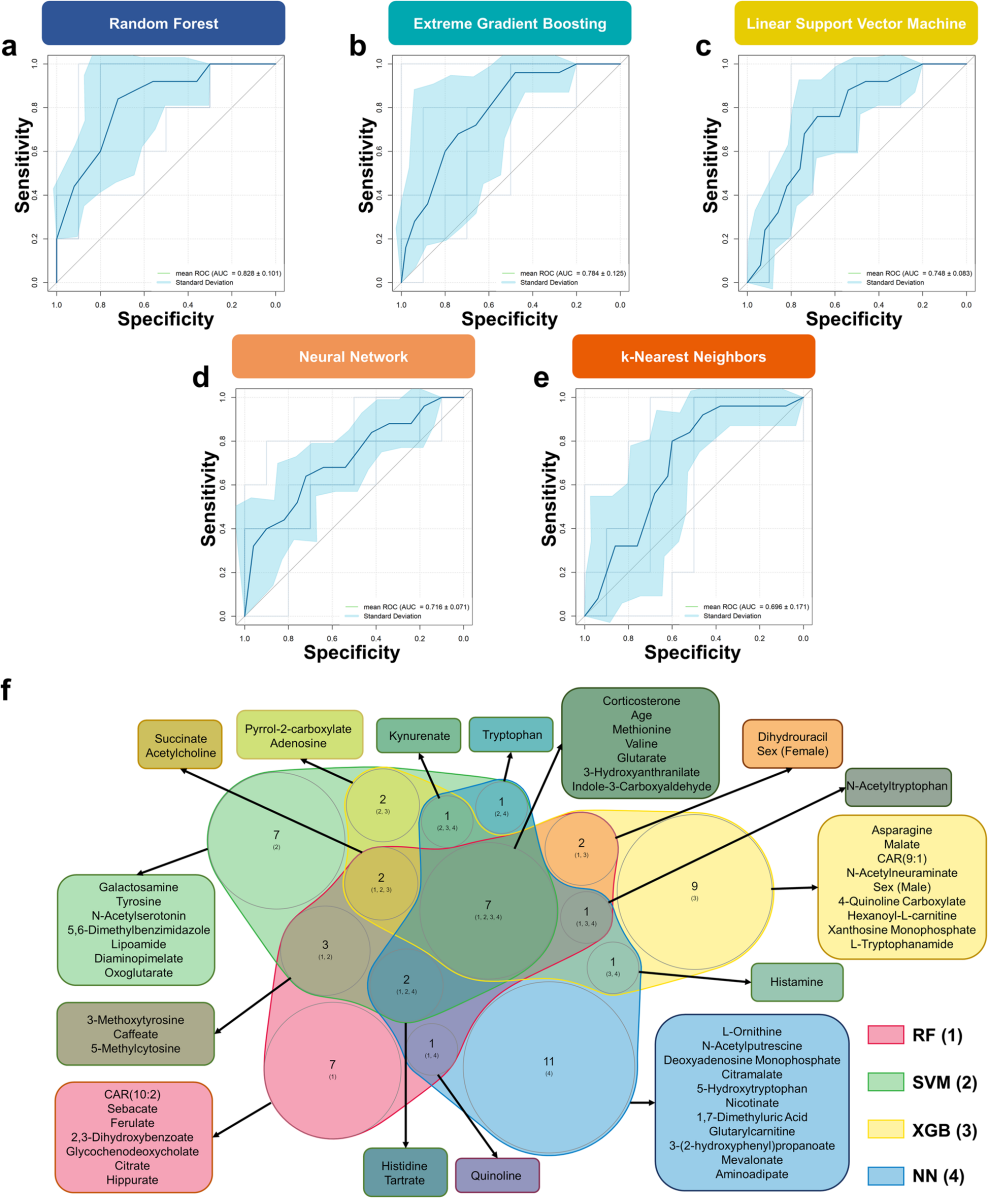
Using machine learning to identify biomarker candidates for NTM and TB classification. (**a**) Receiver operating characteristic curve of the random forest model. (**b**) Receiver operating characteristic curve of the extreme gradient boosting model. (**c**) Receiver operating characteristic curve of the linear support vector machine model. (**d**) Receiver operating characteristic curve of the neural network model. (**e**) Receiver operating characteristic curve of the k-nearest neighbors model. (**f**) Venn analysis between top 10% variable based on importance score of random forest, linear support vector machine, extreme gradients boosting, and neural network models. *NTM* nontuberculous mycobacteria, *TB* tuberculosis, *RF* random forest, *SVM* support vector machine, *XGB* extreme gradient boosting, NN, neural network.

The results from the data exploration and conventional statistical analyses indicated a subtle difference in metabolic profiles between NTM infection and TB. Therefore, we applied ML to better capture the difference between the metabolomes of these two groups. The RF model performed excellently in classifying NTM and TB patients (AUC of ROC curve and standard deviation from the five-fold nested cross-validation = 0.828 ± 0.101) (Fig. 4a). The classification performance of three other models (SVM, XGB, and NN) was acceptable (AUC > 0.7) (Fig. 4b–d). However, k-NN model performed poorly with high variability (AUC = 0.696 ± 0.171) (Fig. 4e). Therefore, the variable importance score estimated from the k-NN model was not considered for biomarker selection.

Venn analysis was conducted between the variables ranked in the top 10% based on the importance scores of SVM, RF, XGB, and NN (Fig. 4f and Supplementary Fig. S5) to identify potential biomarker candidates. There were seven variables that overlapped between the four considered models, including six annotated metabolic features (3-hydroxyanthranilate, corticosterone, glutarate, methionine, valine, and indole-3-carboxyaldehyde) and one demographic variable i.e., age. Moreover, the other six annotated metabolic features were ranked as the top 10% most important variable for three out of five models. These included succinate and acetylcholine (SVM, RF, XGB); histidine and tartrate (SVM, RF, NN); kynurenate (SVM, XGB, NN); N-acetyltryptophan (RF, XGB, NN).

Of note, given that age was significantly different between the two groups, it also appeared to be one of the most important variables for all considered models. To investigate the potential confounding effect of the age variable, we assessed the model performance after removing it from the models. The results showed no statistically significant improvement in performance for all investigated models (Supplementary Fig. S6, Supplementary Table S1). This finding implied a negligible confounding effect of age on model performance. In general, ML showed promising results for distinguishing NTM from TB. In total, we identified 12 annotated metabolic features as potential biomarker candidates for the differential diagnosis of NTM infection.

## Discussion

The prevalence of NTM lung disease has been increasing globally^5^. Misdiagnosis of NTM lung disease versus TB remains a challenge, leading to inaccurate treatment and unfavorable outcomes^48,49^. In this study, we identified several metabolic noninvasive biomarkers that may aid in the differential diagnosis of these two diseases, using untargeted metabolomics and ML. Previous studies have utilized metabolomics to discover metabolic biomarkers for differentiating NTM infection patients from healthy controls^24^, or between NTM-positive and NTM-negative cystic fibrosis patients^50^. However, to our knowledge, this is the first study to explore the potential of urinary metabolic biomarkers for the differential diagnosis of NTM infection versus TB. When considered for clinical application, especially for a triage test, urine samples have several advantages, such as simplicity, comfort, and noninvasiveness in sample collection. Of note, the World Health Organization has endorsed a urine-based assay for TB diagnosis^29^. Here, the application of a rapid data acquisition method showed the potential to measure urinary biomarkers using a fast LC–MS assay, which further facilitates the translation of such biomarkers for clinical application.

ML provides a valuable approach for biomarker discovery^51^. ML is widely employed to select biomarker candidates because of its ability to learn the complex relationships between features and rank them based on their contribution to model performance. However, the prioritization of the “potential biomarkers” varies significantly among ML algorithms and depends considerably on the quality and quantity of the datasets used for training and validation^52,53^. Therefore, the shortlist of potential biomarkers derived from a single ML model might not be generalized well in cross-study validation^53^. Covariates commonly found to be important features among algorithms are expected to have higher translational value. To overcome this disadvantage of using a single ML model, we proposed a list of the most promising biomarker candidates based on the consensus of multiple ML models. Remarkably, typical chemometric modeling (i.e., PCA and PLS-DA) and simple k-NN failed to provide valid classifications due to the high variability in their performance. This finding, along with the unsatisfied results of univariate linear regression method and the promising performance of more sophisticated ML algorithms, implied the complex nature of our data and justified our approach of using ML for biomarker identification to differentiate NTM infection and TB. Of note, our choices for ML were representatives from multiple classes of algorithms, ranging from the naive one (i.e., k-NN) to the state-of-the-art one for numeric data (e.g., neural network). This selection was a way to confirm that there were no simple solutions for TB-NTM differential diagnosis. Among the five ML models used, only the k-NN model displayed invalid classification with high variability of the AUC value. This phenomenon could be explained by the susceptibility of the k-NN model to high-dimensional data, especially in a dataset with a small sample size. Additionally, k-NN may not perform well when the outcome variable is imbalanced^54^.

The current study revealed metabolic alterations in NTM patients compared to TB patients. Amino acids such as methionine were elevated, while valine, histidine, tyrosine, and tryptophan were less abundant. Amino acids play an essential role in the host innate and adaptive immune response to infections^55^. Particularly, the alteration of methionine could be related to the host oxidative stress responses to pathogens^56^. On the other hand, histidine alteration may be caused by a complex crosstalk effect between the host and pathogens^57^. The alterations of amino acids observed in our study may be related to the differences in host immune responses between NTM infection and TB. Interestingly, we detected the reduction of 3-hydroxyanthranilate, an intermediate in the tryptophan metabolism pathway^58^. A study comparing the sputum of NTM-positive and NTM-negative cystic fibrosis patients also found a low level of tryptophan and its metabolite, anthranilate, in the NTM-positive group^50^. Another study in an animal model found a decrease of plasma tryptophan level in the group infected with *Mycobacterium avium*^59^. In contrast, the urinary level of 3-hydroxyanthranilate has been reported to be higher in TB patients compared to healthy individuals. Kynurenate, another metabolite in the tryptophan catabolic pathway, was among the top features of our ML models. The tryptophan catabolism pathway is known to regulate the host immune response to pathogens^60^. Our findings align with previous reports on NTM and may implicate heterogeneous catabolism of tryptophan between NTM infection and TB. Furthermore, indole-3-carboxyaldehyde, a derivative of tryptophan derived from the gut microbiota, was found to suppress IL-6 cytokine production of murine macrophages in response to stimulation by *Mtb*^61^. Previous reports have also correlated the gut microbiome with host immune responses to *Mtb* infection^62^ and NTM infection^63^. The alteration of indole-3-carboxyaldehyde in our study may be partially explained by the role of tryptophan metabolism mediated by gut microbiome in the immune responses to NTM infection and TB.

The age of the NTM patients in our cohort was significantly older than that of the TB patients, which is consistent with previous reports^64,65^. A study showed that age older than 50 years is a potential predictive factor in classifying NTM and TB^64^. This finding aligns with our results, which showed that age appears to be one of the most important features contributing to the classification of NTM and TB for all models considered. The increased risk of NTM infection in elderly individuals may be explained by the decline in immunity with age and a higher likelihood of receiving medical treatments^66,67^.

Some weaknesses of the study due to its exploratory nature and sampling limitations need to be discussed. First, the lack of quantitative concentrations of urinary metabolites in our study may hamper the direct translation of these biomarkers in clinical settings. A targeted absolute quantification assay is needed to facilitate the clinical application of these biomarkers. Second, given that the sample size of our study was small, no external validation could be performed. However, our robust nested cross-validation could be sufficient to ensure the validity of the biomarker candidates and warrants further investigation. Inter-cohort cross-validation with a larger sample size and more diverse population is still required to fine-tune the signature and ensure its generalizability. Finally, it is of potential interest to examine the role of the biomarker candidates by including other groups, such as healthy controls and patients with cystic fibrosis. It may help expand the usability of our assay beyond the differential analysis of NTM and TB. It is worth mentioning that diagnostic biomarkers might also be applied for other purposes, such as predicting the risk of disease progression or monitoring treatment^28^. A multiple-purpose signature could be employed to comprehensively monitor the status of patients from infection to treatment, advancing personalized medicine for NTM infection^68^. Hence, the capacity of our biomarkers in these applications should be evaluated in future studies.

## Conclusion

In conclusion, we applied a fast LC–MS untargeted metabolomics approach to discover urinary biomarkers for diagnosing NTM pulmonary disease. Between NTM infection and TB, metabolic alterations in amino acid levels and tryptophan metabolism were revealed. These findings implicate the differences in host immune response and host–pathogen metabolic crosstalk between these two *Mycobacteria* infections. Using univariate and multivariate analyses, incorporating ML algorithms, we identified several potential biomarkers for differentiating between NTM infection and TB, including valine, corticosterone, glutarate, 3-hydroxyanthranilate, and indole-3-carboxyaldehyde. These biomarkers may aid the accurate diagnosis of NTM infection versus TB. Our ML strategy provided a robust approach to prioritize biomarker candidates in a scenario where the data have a complex underlying structure and small sample size. In addition, integrating fast LC–MS method and ML modeling demonstrate the potential of a semi-automated platform for convenient and scalable diagnostics. Altogether, our study can serve as a foundation for facilitating the use of urinary biomarkers and ML in diagnosing NTM pulmonary disease.

### Supplementary Information

Supplementary Information 1.

﻿Supplementary Information 2.

## Data Availability

Data is provided within the supplementary information file.
