# Supplementary material for: Discovery of urinary biosignatures for tuberculosis and nontuberculous mycobacteria classification using metabolomics and machine learning
Source: Sci Rep. 2024 Jul 3;14:15312. doi: 10.1038/s41598-024-66113-x (PMC11222504; doi:10.1038/s41598-024-66113-x)
Supplement: Supplementary file 2 — ﻿Supplementary Information 2. [file 41598_2024_66113_MOESM2_ESM.pdf]

Discovery of Urinary Biosignatures for Tuberculosis and Nontuberculous Mycobacteria Classification Using Metabolomics and Machine Learning

Nguyen Ky Anh[1,2,#], Nguyen Ky Phat[1,#], Nguyen Quang Thu[1], Nguyen Tran Nam Tien[1], Cho Eunsu[1], Ho-Sook Kim[1], Duc Ninh Nguyen[3], Dong Hyun Kim[1], Nguyen Phuoc Long[1,*], Jee Youn Oh[4,*]

[1]Department of Pharmacology and PharmacoGenomics Research Center, Inje University College of Medicine, Busan 47392, Republic of Korea

[2]Faculty of Pharmacy, Ton Duc Thang University, Ho Chi Minh City, Vietnam

[3]Section for Comparative Pediatrics and Nutrition, Department of Veterinary and Animal Sciences, University of Copenhagen, Frederiksberg 1870, Denmark

[4]Division of Pulmonary, Allergy and Critical Care Medicine, Department of Internal Medicine, Korea University Guro Hospital, Seoul 08308, Republic of Korea

*: Corresponding to: Nguyen Phuoc Long (phuoclong@inje.ac.kr) and Jee Youn Oh (happymaria0101@hanmail.net).

#: These authors contribute equally on this work.

Supplementary Fig. S1. Principal component analysis scores plots of urine metabolome that contained quality control samples. (**a**) Positive Ion Mode. (**b**) Negative Ion Mode.

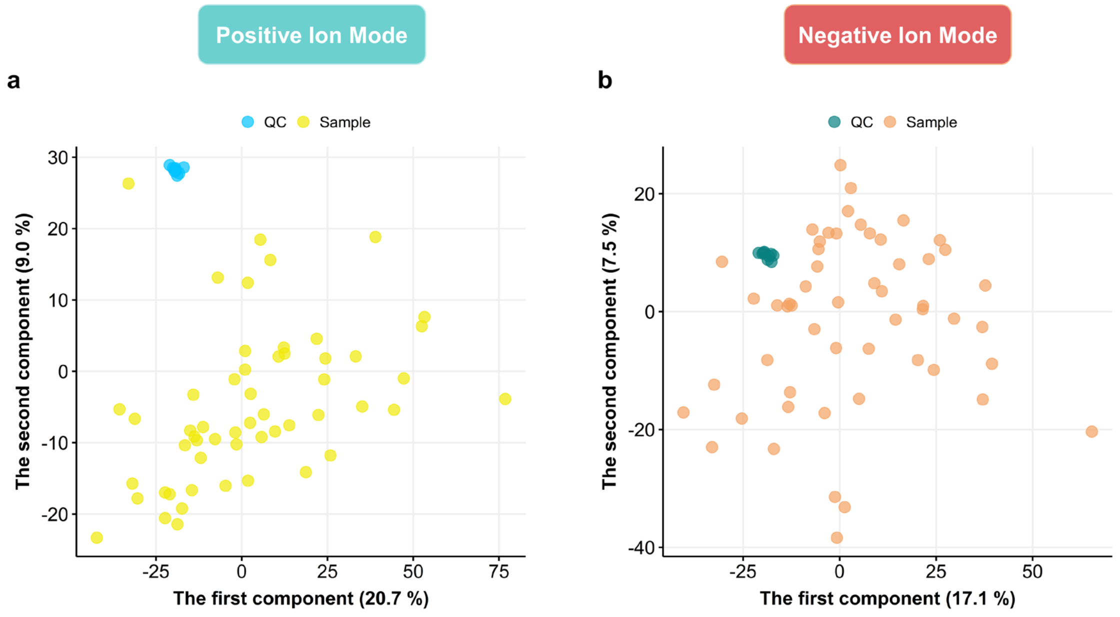

Supplementary Fig. S2. Partial least squares – discriminant analysis cross-validation results. (**a**) Positive Ion Mode. (**b**) Negative Ion Mode.

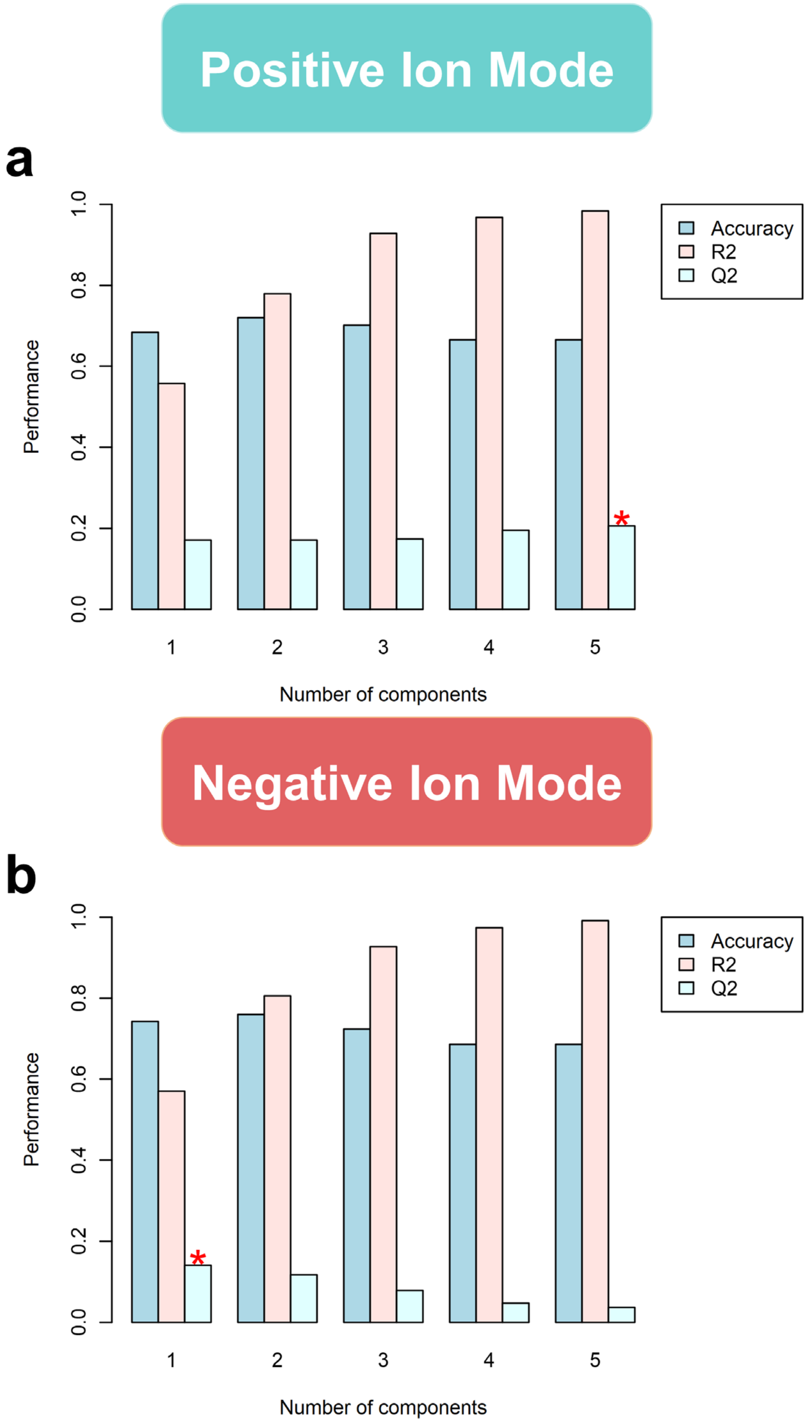

Supplementary Fig. S3. Volcano plot of differential metabolic features between NTM and TB patients. (**a**) Positive ion mode with P-value and fold change cut-off. (**b**) Positive ion mode with false discovery rate and fold change cut-off. (**c**) Negative ion mode with P-value and fold change cut-off. (**d**) Negative ion mode with false discovery rate and fold change cut-off. Abbreviations: FC, fold change; FDR, false discovery rate.

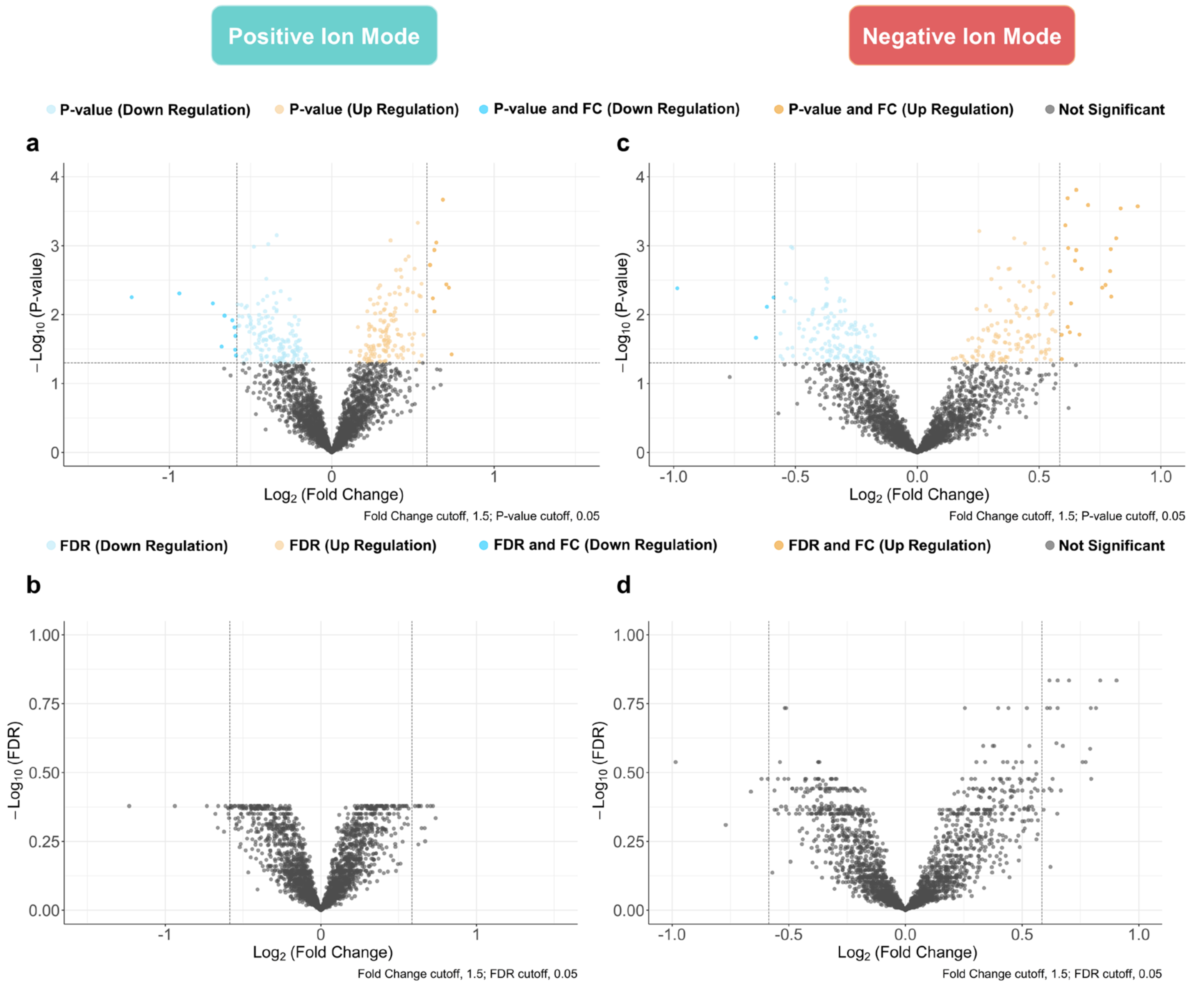

Supplementary Fig. S4. Covariates plot of linear models. (a) P-values of pre- and post-adjustment using linear models for positive ion mode. **(b)** FDRs of pre- and post-adjustment using linear models for positive ion mode. **(c)** P-values of pre- and post-adjustment using linear models for negative ion mode. **(d)** FDRs of pre- and post-adjustment using linear models for negative ion mode.

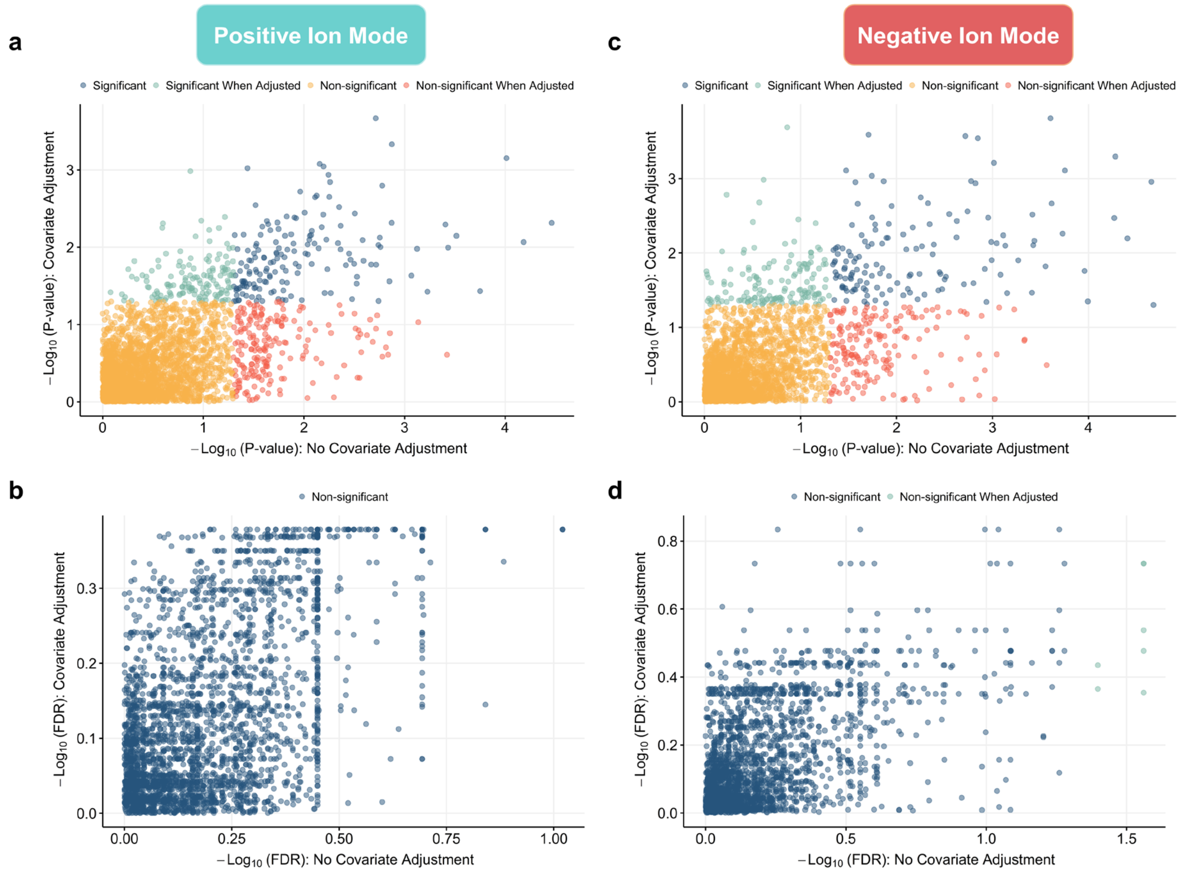

Supplementary Fig. S5. Top 10% variables based on importance score of NTM and TB patient's classification models used for biomarker identification. (**a**) Random forest. (**b**) Linear support vector machine. (**c**) Extreme gradient boosting. (**d**) Neural Network.

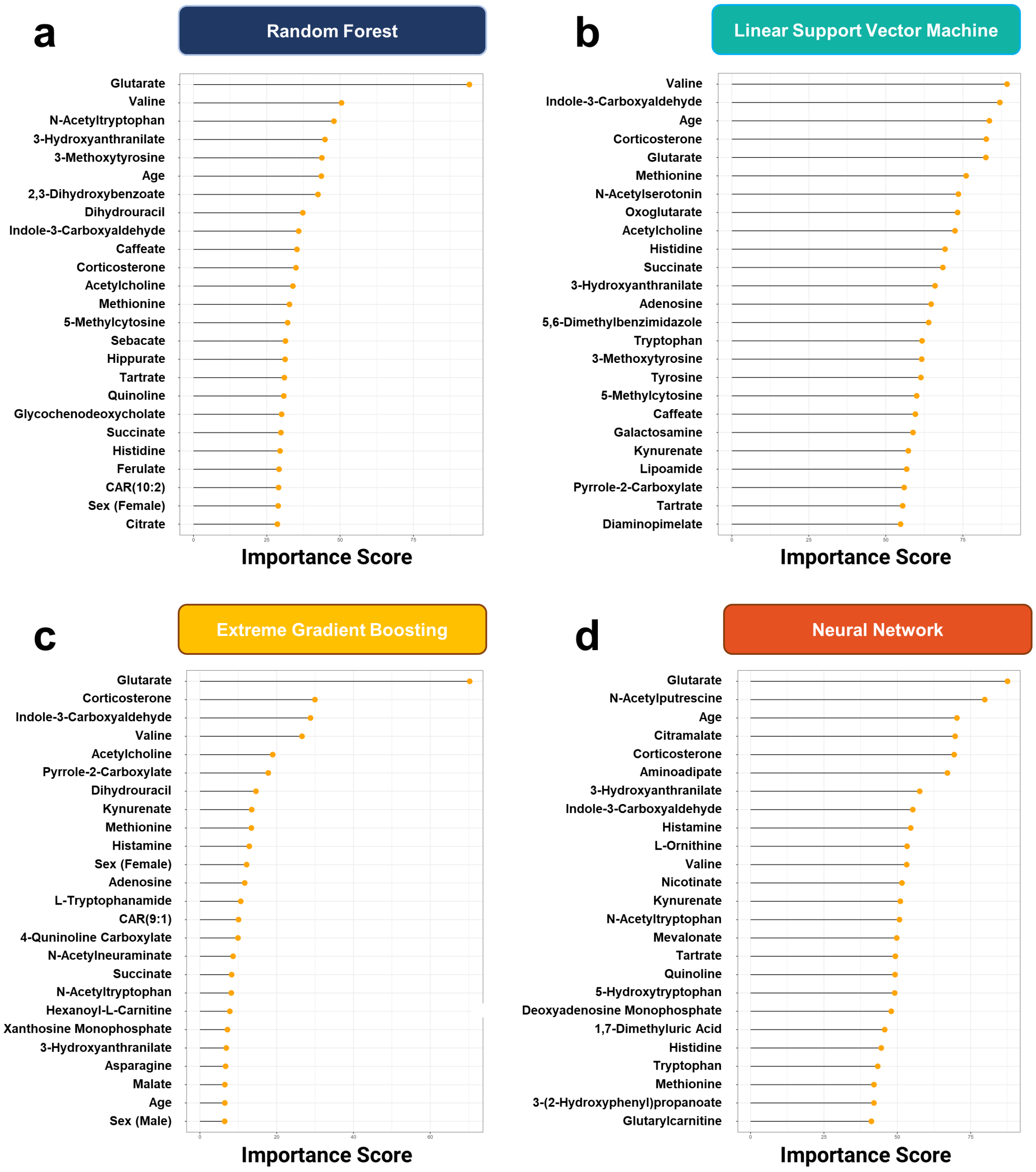

Supplementary Fig. S6. Receiver operating characteristic curve of the models after removing the age variables. **(a)** Random forest model. **(b)** Extreme gradient boosting model. **(c)** Linear support vector machine model. **(d)** Neural network model. **(e)** k-Nearest neighbors model.

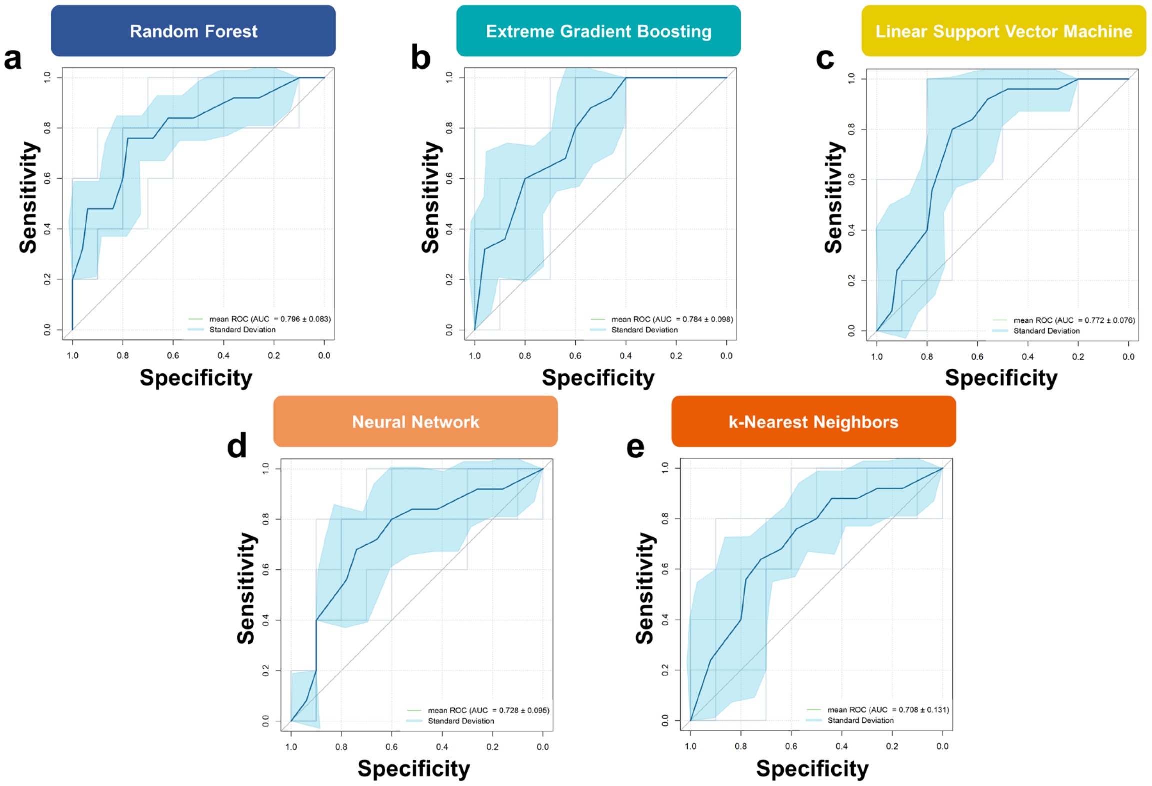

Supplementary Table S1. Performance comparison between models with and without age variables.

Model	AUC ± SD		P-value#
	**With Age**	**Without Age**	**(Two-sided Wilcoxon Rank Sum Test)**
Random forest	0.828 ± 0.101	0.796 ± 0.083	0.691
Extreme gradient boosting	0.784 ± 0.125	0.784 ± 0.098	0.753
Linear support vector machine	0.748 ± 0.083	0.772 ± 0.076	0.834
Neural network	0.716 ± 0.071	0.728 ± 0.095	1.000
k-Nearest neighbors	0.696 ± 0.171	0.708 ± 0.131	0.917

AUC: Area under the receiver operating characteristic curve

SD: Standard deviation

Significant threshold was P-value < 0.05
